# Combined Effect of High Hydrostatic Pressure, Sous-Vide Cooking, and Carvacrol on the Quality of Veal, Plant-Based, and Hybrid Patties during Storage

**DOI:** 10.3390/foods12020289

**Published:** 2023-01-08

**Authors:** Rasmi Janardhanan, Carmen Olarte, Susana Sanz, Carmina Rota, María José Beriain

**Affiliations:** 1 Institute for Innovation & Sustainable Food Chain Development, Universidad Pública de Navarra, Campus de Arrosadía, 31006 Pamplona, Spain; 2 Departamento de Agricultura y Alimentación, Universidad de La Rioja, 26006 Logroño, Spain; 3 Departamento de Producción Animal y Ciencia de los Alimentos, Veterinary Faculty, Universidad Zaragoza, 50013 Zaragoza, Spain

**Keywords:** high-pressure processing, beef, meat analog, textural property, microbial analysis

## Abstract

The effect of carvacrol added to patties stored at 4 °C for 14 days, previously pressurized and vacuum-cooked (HPP-SVCOOK), was investigated. Three formulations were prepared (veal, plant-based product, and hybrid product). An emulsion made with olive and linseed oils was added. The physicochemical and microbiological qualities were assessed. Microbial tests indicated negligible growth of spoilage organisms in treated patties. No significant effect of carvacrol on the microbial loads of patties was noticed. Sulfite-reducing clostridia and Enterobacteriaceae were absent in the treated patties, whereas, in the treated veal and hybrid samples, 3 and 2 units of log cfu/g reduction for lactic acid bacteria and molds and yeasts were noted, respectively. On day 7 of storage, veal patties exhibited a significant reduction (*p* < 0.05) in the L* (53.9–49.3), hardness (32.3–21.4 N), springiness (0.8–0.7 N), cohesiveness (0.49–0.46), and chewiness (12.2–7.1) and a hike in the a* value (5.3–9.4). No significant changes in L* (59.1–58.6), a* (8.57–8.61), hardness (11.6–10.6 N), or cohesiveness (0.27–0.26) were observed in plant-based patties over the storage times, whereas reductions in springiness (0.5–0.4), chewiness (1.9–1.3), and b* (26.6–29.1) were noted in them. In hybrid patties, the L* (53.9–52.5) and b* values (24.9–24.3) were consistent but had a significant decrease in a* value (5.9–3.5) along the days of storage under study. The texture parameters of the hybrid patties altered were similar to those of veal patties during the 14-day storage time. In all samples, pH decreased with storage time. HPP-SVCOOK was effective on rendering safe and shelf-stable, ready-to-eat patties regardless of their matrix formulation. The addition of carvacrol had limited effects on the textural qualities of the HPP-SVCOOK products. Future studies need to be undertaken to assess the treated patties’ consumer acceptability and sensory profile. The study provides the basis for the development of novel meat-based and plant-based products that are microbiologically safe, with minimum physicochemical alterations during storage.

## 1. Introduction

Currently, there is an increase in the acceptability and demand for alternative protein due to the perception of it being more sustainable [1,2]. According to various survey reports, 41% Gen Z and Millennials adopt plant-based products at restaurants [3]. High carbon footprints, environmental stress, and animal welfare issues have induced diet changes [4,5]. These multifaceted issues have pressed the food industry to look for alternative protein sources [6]. Many studies have been carried out so that innovative products similar to meat can be produced [7,8,9]. In 2022, soy protein has been estimated as the largest segment contributing to the plant protein market [10]. In addition, a growing consumer preference for hybrid products (i.e., meat blended with plant protein sources) has been noted [11]. This change in mindset and strategy to cater to the evolving consumer preferences and demand has led to the expansion of the alternative protein industry [6]. 

Processed meat generally refers to meat preserved by nitrite curing, drying, smoking, or added with food additives with potential health risks [12,13]. The polycyclic aromatic hydrocarbons and heterocyclic amines formed in meat during heat treatment at high temperatures have the ability to damage DNA [13]. Meat products are known to be high in saturated fats and cholesterol [14]. Some epidemiological studies have pointed out that consumption of meat products beyond the dietary recommendations might be associated with cardiovascular illnesses and cancer [15]. Microencapsulated PUFA-rich oils have been used to prepare healthy beef burgers [16]. Fat replacement has been used as a strategy for the preparation of healthy dry fermented sausages [17]. Janardhanan et al. [18,19,20] reported that the combination of emerging technologies coupled with healthy fat replacement could be a successful strategy in the preparation of clean-label, sustainable, and novel meat and alternative protein products. High-pressure processing (HPP) is one of the emerging technologies currently used in the food industry to preserve food with minimal alterations [21,22,23]. Numerous researchers have noted the advantages of HPP in preserving flavor compounds and the nutritional value of meat products while extending the shelf life [24,25,26]. Meat subjected to HPP retains its organoleptic properties and can be positively described as a “clean-label” food product [27,28]. Sous vide, also referred as low-temperature, long-time (LTLT) cooking (SVCOOK), is a cooking method used for the preparation of high-quality dishes [25]. The food is vacuum-packaged and immersed in hot water at a lower temperature than the normal cooking temperature. The temperature is monitored and maintained throughout the process. Lower cooking loss and lipid oxidation with simultaneous color and flavor enhancement are reported advantages of cooking meats using SVCOOK [25,29]. 

Sulfites, nitrites, phosphates, and other food additives have been used as preservatives due to their antioxidant and antimicrobial properties [30,31]. In the EU, sulfite in meat and meat products is restricted to burger meat and breakfast sausages. Natural antimicrobials can replace their conventional analogs and fulfill the consumer demand for “clean-label” foods [32]. Carvacrol is one of the monoterpene phenols found in the essential oils of aromatic plants such as thyme and oregano [33]. Several studies on minimal inhibitory concentration or minimal bactericidal concentration concluded the biostatic and biocidal property of carvacrol against bacteria and fungi [30,33,34,35,36,37]. Its bactericidal property is attributed to the antimicrobial agent’s effect on structural and functional properties of the cytoplasmic membrane [38]. The effects of the combined application of HPP and SVCOOK on the physicochemical and microbiological properties of raw and processed meat have been reported [18]. The possibility of using the combined HPP-SVCOOK technology for the elaboration of safe and sustainable meat products was recommended [18]. It was hypothesized that the dual application of HPP and SVCOOK on patties prepared with different protein matrices would ensure microbiological safety with a minimal impact on the intrinsic physicochemical characteristics throughout conventional storage periods. Therefore, this experiment aimed to investigate the extent to which the combined effect of HPP, SVCOOK, and the addition of an antimicrobial agent (carvacrol) affects the quality of veal, plant-based, and hybrid patties during storage. 

## 2. Materials and Methods

### 2.1. Sample Preparation

All the raw materials were locally procured. The study was conducted on patties of three different formulations, i.e., veal, plant-based, and hybrid. The samples were prepared as described by Janardhanan et al. [20]. The plant-based ingredient was *Legumbreta fina*. The meat (*Biceps femoris*) used for the preparation of the meat-based and hybrid patties were from Ternera de Navarra. The hybrid patty was prepared with a 50-50 protein mix of meat and plant-based products. The soy-based oil-in-water emulsion used as the fat replacer was prepared according to Janardhanan et al. [19].

The protein matrix (78.5%), emulsion (20%), salt (1.5%), and Provençal herbs (0.01%) were added and blended to reach uniformity. After the addition of the emulsion, two batches were prepared: one batch was added with 150 ppm of the antimicrobial agent, carvacrol [30] (98%, Sigma-Aldrich, Merck Life Science S.L.U. Madrid, Spain). Authors have reported a positive antimicrobial effect of carvacrol at 300 ppm concentration in lamb patties, but it had an adverse effect on the sensory and organoleptic properties [30]; therefore, a lower level of carvacrol was selected. Samples were pressed into patties of 150 g each and vacuum-packaged (98%) using a chamber vacuum machine (C412 Lerica, Venice, Italy). The patties were stored at 4 °C overnight before further treatment. 

### 2.2. Experimental Design

**Figure 1:**
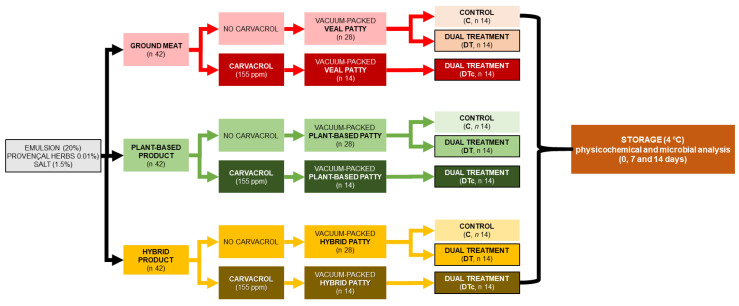
Experimental design for preparing veal, plant-based, and hybrid patties by high-hydrostatic-pressure processing (HPP) and sous-vide cooking (SVCOOK), or dual treatment (DT), and carvacrol (c).

A full factorial-completely randomized variable design was used, and the whole experiment was replicated twice (Figure 1). Patties were prepared with the three different protein matrix formulations, and these were subjected to three treatments that included unprocessed (control), dual treatment of HPP and SVCOOK (HPP-SVCOOK), and carvacrol-added HPP-SVCOOK (HPPSVAA) samples. The experimental design consisted of two batches wherein three individual patties per formulation were HPP and HPPSVAA processed, and two individual patties per formulation were HPPSVCOOK processed in each batch. A total of one hundred and twenty-six patties were prepared, forty-two patties of each protein matrix. The number of patties (*n* = 14) required for the nine different treatments were prepared separately and individually vacuum-packaged. All the patties except the raw (control) samples were HPP-treated and subsequently cooked (SVCOOK) on the same day. The HPP-SVCOOK treatment conditions were selected based on the response surface methodology optimization conducted in *Biceps femoris* beef patties [18]. At the optimized condition, the absence of *Salmonella* species and *Listeria monocytogenes* was reported. The *Escherichia coli* count was presented to be below acceptable limits [18]. Thereafter, all samples were stored at 4 °C until further physicochemical analysis and shelf-life studies. The physicochemical and microbiological analyses of the samples were conducted at 0, 7, and 14 days of storage (Figure 1).

### 2.3. Treatments

The samples were pressurized at 350 MPa for 10 min and subsequently vacuum-cooked at 55 °C as described by Janardhanan et al. [18]. The samples were stored at 4 °C until further analysis.

### 2.4. Proximate Analysis

Moisture [39], protein [40], fat [41], and total ash contents [42] of the plant-based, veal, and hybrid samples of the control and HPPSVAA were performed in triplicates. The prepared patties were vacuum-packaged and frozen (20 days); the samples were thawed at 4 °C, 24 h before the experiments. All the analyses were conducted on ground samples. Moisture content was determined using oven drying until constant weight of a 5 g test sample at 102 °C; protein content was determined using the classical macro-Kjeldahl method with 6.25 as the conversion factor for meat, hybrid [40], and plant-based samples [43]; fat content was determined using a Soxhlet apparatus by petroleum ether extraction; ash content was determined by combusting a 5 g test sample in a muffle furnace at 550 °C until white to light grey residues appeared. 

### 2.5. pH

The pH of the samples was measured in quintuplicates at 25 °C employing a pH-meter (Crison Instruments S.A., Barcelona, Spain) with a combined probe electrode [44]. The device was calibrated using pH buffer solutions of pH 4.01 and 7.00 at 25 °C. 

### 2.6. Instrumental Color

Color parameter (L^*^, a^*^, and b^*^) values were collected in quintuplicates. A handheld spectrophotometer (Minolta 2300d, Konica Minolta Business Technologies Inc., Tokyo, Japan) was used for measuring the color parameters using a D65 illuminant with a 52 mm diameter sphere size, 8 mm measurement area, and 10° observer angle. The instrument was zero and white-calibrated before use.

### 2.7. Instrumental Texture 

Texture parameters were determined according to the method described by Mittal et al. [45]. A Texture Profile Analysis (TPA) of the samples was conducted using a texture analyzer (TA-XT2i, Stable Micro Systems Ltd., Surrey, UK) fitted with a loadcell of 30 kg. Samples (1.5 × 1.5 cm) were subjected to a two-cycle 50% compression at room temperature. The compression time was set as 3 s. A 25 mm aluminum cylindrical probe with a pre-test, test, and post-test speed fixed at 2 mm/s was used. Data from eight consecutive measures were collected with Exponent Lite version 6.1. software (Stable Micro Systems Ltd., Surrey, UK). The hardness (N), springiness (N), cohesiveness, and chewiness were studied.

### 2.8. Microbial Analysis

The samples were aseptically weighed (25 g) and homogenized for 2 min with 225 mL of sterile soy peptone water (0.1% soy peptone plus 0.5% sodium chloride) using a stomacher (IUL, Barcelona, Spain) to obtain a first dilution of 1:10. The absence of colonies in a plate was assessed as <10 colony forming units (cfu)/g (<1 log cfu/g). Further decimal dilutions were made with the same diluent. Lactic acid bacteria were enumerated on MRS Agar medium (pH 6.2 ± 0.2 at 25 °C, Difco, Detroit, MI, USA) following the pour plate method and incubated at 31 °C ± 1 °C for 48 h. [45]. Enterobacteriaceae were determined in VRBG medium (Difco, Detroit, USA) overlayed with the same medium and incubated at 37 °C ± 1 °C for 24 h [46]. Czapek Dox Agar medium (Scharlab S.L., Barcelona, Spain) was employed for molds and yeast enumeration, and plates were incubated at 31 °C ± 1 °C for 48 h [46]. To enumerate the sulfite-reducing clostridia, TSN Agar medium (Scharlab S.L., Barcelona, Spain) and the pour plate method were employed. Plates were overlayed with the same medium and incubated at 45 °C ± 1 °C for 24 h. [46]. All the analyses were performed in duplicate. 

### 2.9. Statistical Analysis

Descriptive statistics for the physicochemical parameters of the control, HPP-SVCOOK, and HPPSVAA samples were calculated. Data analysis and modeling were conducted using Minitab software (Minitab^®^ version 19.2020.1, Minitab LLC., State College, PA, USA).

A mixed-effect model was used to study the effect of different formulations on the physicochemical properties. Replication was added as a random effect in studying the physical parameters. A multiple comparison test was conducted using *post hoc* Tukey analysis at a 95% confidence interval (*p* < 0.05). The mixed-effect model was used to identify the significant effect of the fixed terms (treatment and storage days), and their interaction on the physicochemical parameters.

## 3. Results and Discussion

### 3.1. Proximate Analysis

The moisture, protein, fat, and ash contents of the samples are presented in Table 1. All the formulations had similar protein and fat content. According to their proximate composition, these products could be nutritionally labeled as low-fat patties [18,47]. The lower moisture content in the plant-based samples could be attributed to the compositional profile of the raw material used herein. 

### 3.2. Effect of Treatments and Storage Time on pH

pH values for control and treated veal samples were significantly different (*p* < 0.05) on day 0. In contrast, on the 7th and 14th days, no significant difference (*p* > 0.05) was observed between the HPP-SVCOOK and HPPSVAA samples. Similar results were also observed for the hybrid samples. In the case of plant-based samples, there was no significant difference in pH, due to the treatments except for the raw samples on days 7 and 14; similarly, no significant effect of HPP-SVCOOK was noted in a previous study [20]. However, all formulations (veal, plant-based, and hybrid samples) had a significant reduction in pH during the first 7 days of storage (Table 2). 

In meat and meat products, a variation in pH may occur due to protein denaturation, myofibrillar lattice spacing, and shrinkage as a result of cooking and HPP treatment [48]. An increase in pH due to protein denaturation has been reported previously [49,50]. The combination of HPP and SVCOOK may cause denaturation of the protein, leading to an increase in pH. A rise in pH during the first 15 days of storage of HPP-treated minced beef (350 MPa, 10 min, stored at 4 °C) [49] and until 30 days of storage in beef capriccio (450 MPa, 5 min, stored at 8 °C) [51] was reported. A similar decline in pH to that observed in the present study was noted in raw, minced beef [50]. The decrease in pH may be attributable to microbial growth in the products [52].

### 3.3. Effect of Treatments and Storage Time on Texture Parameters

#### 3.3.1. Veal Samples

**Figure A1:**
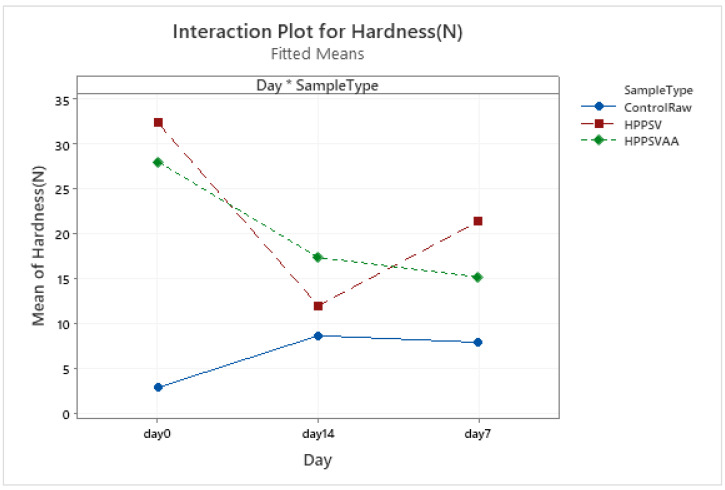
Effect of treatments and storage time on the hardness of veal patty (HPPSV: HPP-SVCOOK).

**Figure A2:**
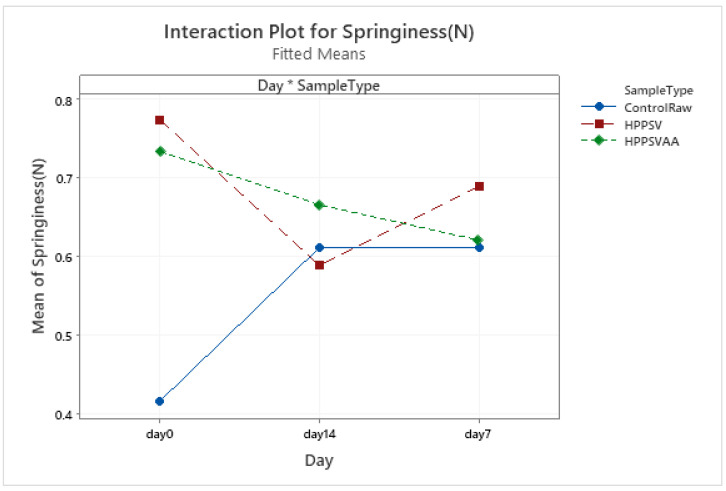
Effect of treatments and storage time on the springiness of veal patty (HPPSV: HPP-SVCOOK).

**Figure A3:**
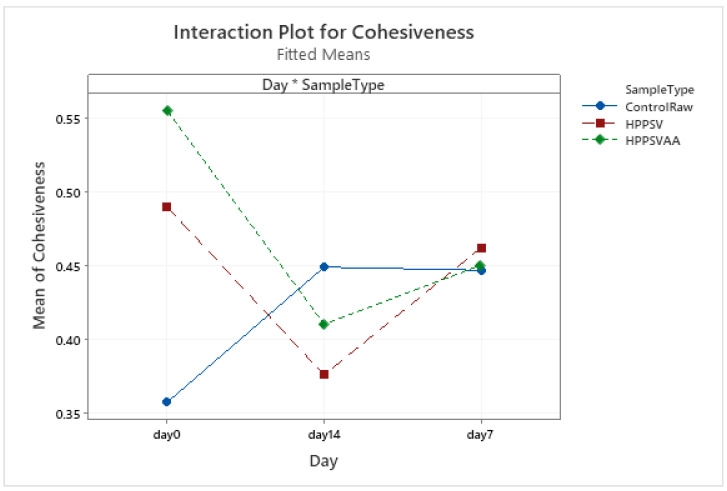
Effect of treatments and storage time on the cohesiveness of veal patty (HPPSV: HPP-SVCOOK).

**Figure A4:**
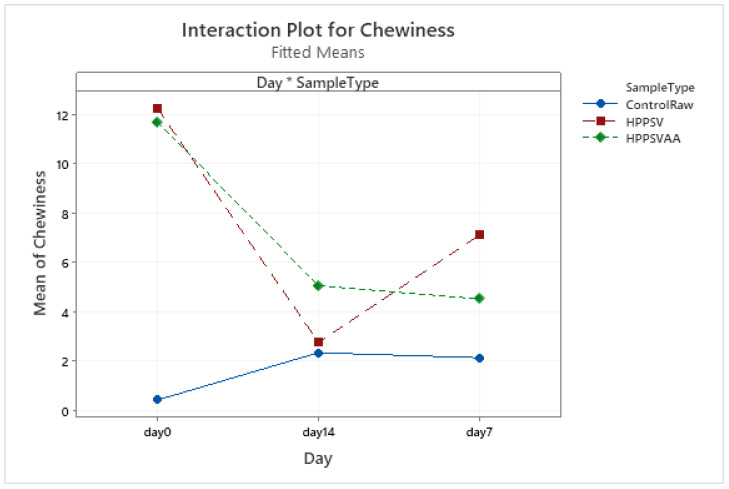
Effect of treatments and storage time on the chewiness of veal patty (HPPSV: HPP-SVCOOK).

HPP-SVCOOK and HPPSVAA samples had significantly higher texture parameter values compared to the control on the 0th day (Table 3). Significant increases in the control samples’ texture parameters were observed in the first week of storage (Figure A1, Figure A2, Figure A3 and Figure A4). In the case of treated samples, values for the texture parameters declined over the first week (*p* < 0.05). The addition of carvacrol did not produce any noticeable effect on the texture of the veal patties.

The hardness decrease in pressurized samples might be attributed to the incomplete inactivation or reactivation of proteolytic enzymes [53]. The increase in hardness and gumminess of HPP-treated meat compared to those of the control had been reported [54]. This might be due to the reduction in the acid, alkaline, and neutral proteases’ activities and the denaturation of actin and sarcoplasmic proteins at pressures of 200–400 MPa [55]. High pressure has the potential to affect the integrity of lysosome and induce a spike in the cathepsin D and acid phosphate activities in pressurized beef throughout the storage, which affect the texture parameters [56]. In chicken breast fillets, chewiness, gumminess, cohesiveness, and hardness increased with pressure (HPP) [57]. Decreases in texture parameters over storage were previously reported [50].

The reduction in the value of texture parameters over the storage time might be due to biochemical and physicochemical changes in meat. This could be due to the natural ageing process reduced by pressurization [56]. Furthermore, a pH lower than 6 as observed in the current study during storage improves the activity of acidic protease, which reduces the hardness [58]. The higher microbial counts (Section 3.5) found in the control samples might have also contributed in the same. 

#### 3.3.2. Plant-Based Samples

The hardness of the control plant-based samples increased until the 7th day with a steady decline over the next week. For the treated samples, no significant change was observed over the storage days. On the 0th day, HPP-SVCOOK samples had a significantly higher hardness (*p* < 0.05). The effect of the carvacrol was prominent in the springiness of the plant-based samples. A slight rise in cohesiveness was observed in the control raw samples along the storage days. Conversely, in the case of treated samples, no significant effect of storage days was observed. No significant effect of the antimicrobial agent along the storage days was observed in chewiness of the samples, but the trends differed slightly between treatments. 

**Figure A5:**
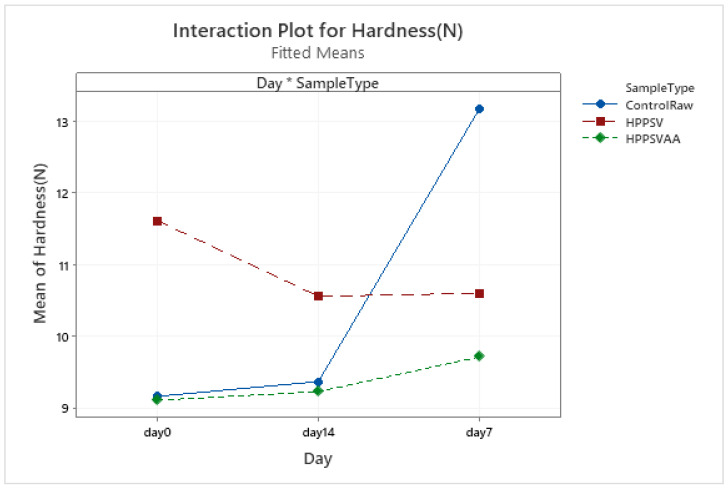
Effect of treatments and storage time on the hardness of plant-based patty (HPPSV: HPP-SVCOOK).

**Figure A6:**
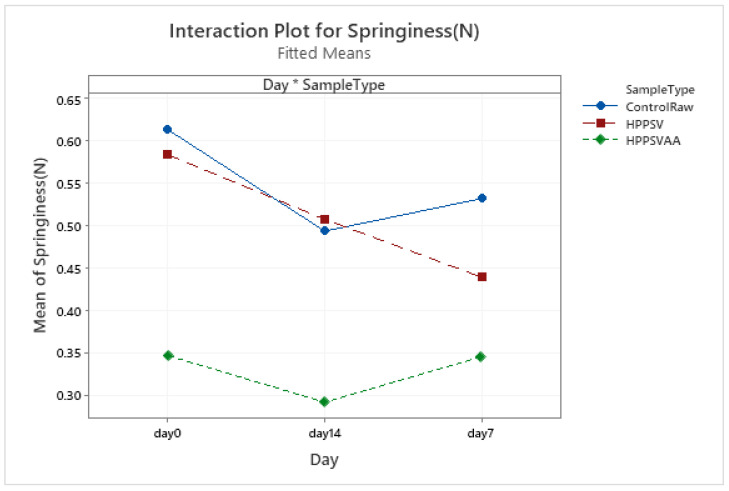
Effect of treatments and storage time on the springiness of plant-based patty (HPPSV: HPP-SVCOOK).

**Figure A7:**
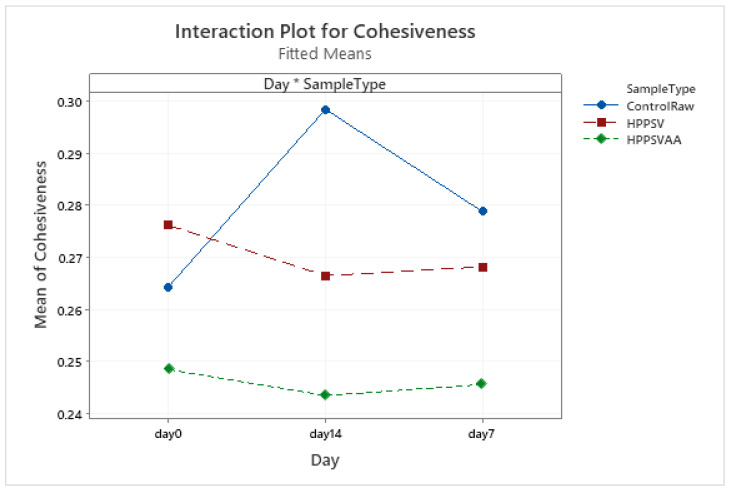
Effect of treatments and storage time on the cohesiveness of plant-based patty (HPPSV: HPP-SVCOOK).

**Figure A8:**
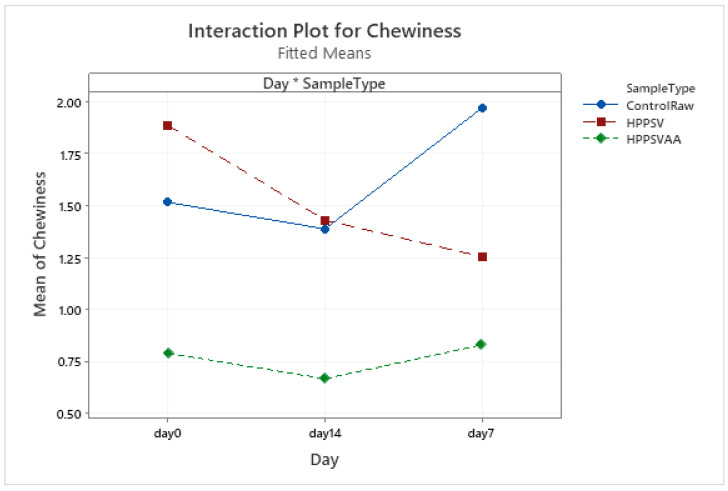
Effect of treatments and storage time on the chewiness of plant-based patty (HPPSV: HPP-SVCOOK).

No specific trend in differences between the control and treated samples could be seen in the plant-based samples (Figure A5, Figure A6, Figure A7 and Figure A8). Except for hardness and springiness, no significant effect of the HPP-SVCOOK or the antimicrobial agent was recorded in the texture parameters (Table 3). Similar results were found in a previous study in our laboratory [20], where the textural profile of plant-based patties differed from that of veal patties. A minimal effect of storage on the texture properties of the plant-based samples might be a result of the use of extruded plant-based raw material for the patty preparation. The extrusion process has the potential to inactivate enzymes such as lipoxygenases activities, resulting in the current observations [59]. The significant increase in hardness in the control samples with the increase in storage days similar to the change observed in veal samples might be a result of their higher microbial counts. When chickpeas were subjected to HPP (200–600 MPa, 1–5 min), a significant reduction in texture parameters was observed with a minimum firmness value at 600 MPa [24]. However, other authors have reported an increase in firmness over a pressure of 600 MPa in chickpeas [60], which was attributed to protein aggregation [61]. A heterogeneous protein denaturation curve in HPP heat-treated (600 MPa for 4 min, 95 °C for 15 min) pea lentil and faba-bean proteins has been reported [62]. This observation was attributed to different sensitivities to HPP and heat because of differences in protein conformation [63]. HPP and heat treatments are known to induce gel network formation at high concentrations of protein [62,63,64]. 

#### 3.3.3. Hybrid Samples

Hardness of the control samples increased (*p* < 0.05) during the first week of storage. Treated samples were significantly harder (*p* < 0.05) than the control on the 0th day. Along the storage, a decline in the hardness of HPP-SVCOOK samples was observed but did not reach statistical significance (Table 3). After the first week of storage, hardness values of the HPPSVAA samples remained similar to the 0th day. An initial increase in the hardness of SPI-incorporated buffalo meat emulsion sausage was followed by a decline after the 14th day, which was inferred to be due to ripening and microbial growth [65]. 

The changes in springiness, cohesiveness, and chewiness of the control samples followed a similar trend to those observed in hardness. No significant difference between the springiness of HPP-SVCOOK and HPPSVAA samples was detected. No significant difference (*p* < 0.05) in the texture parameters due to the added antimicrobial agent was noted along the days. 

**Figure A9:**
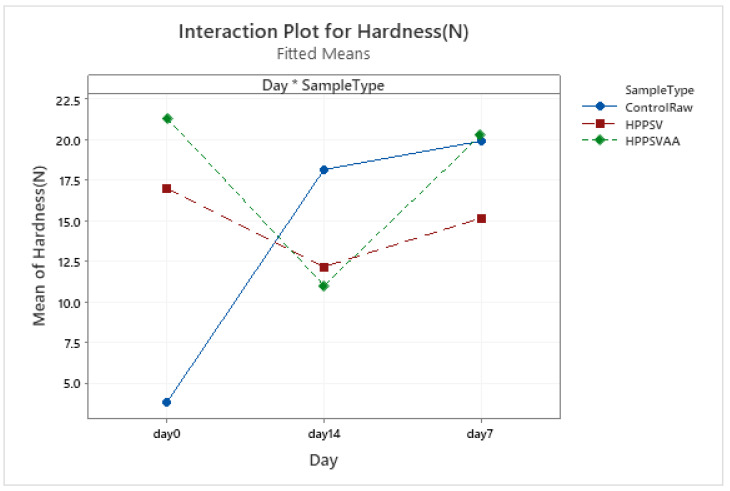
Effect of treatments and storage time on the hardness of hybrid patty (HPPSV: HPP-SVCOOK).

**Figure A10:**
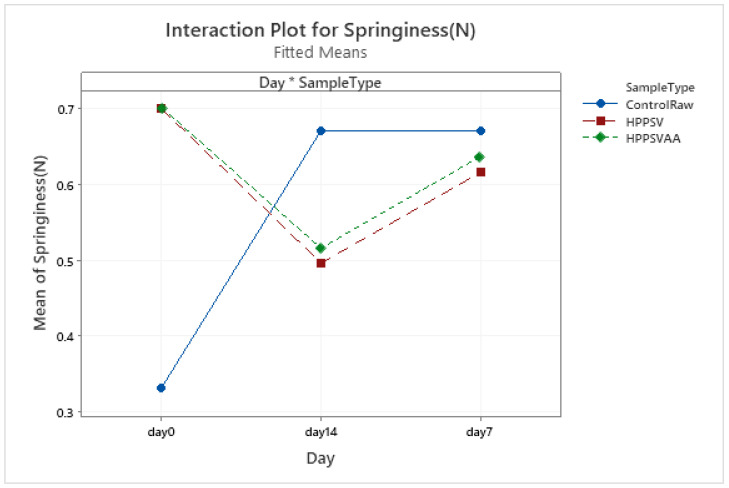
Effect of treatments and storage time on the springiness of hybrid patty (HPPSV: HPP-SVCOOK).

**Figure A11:**
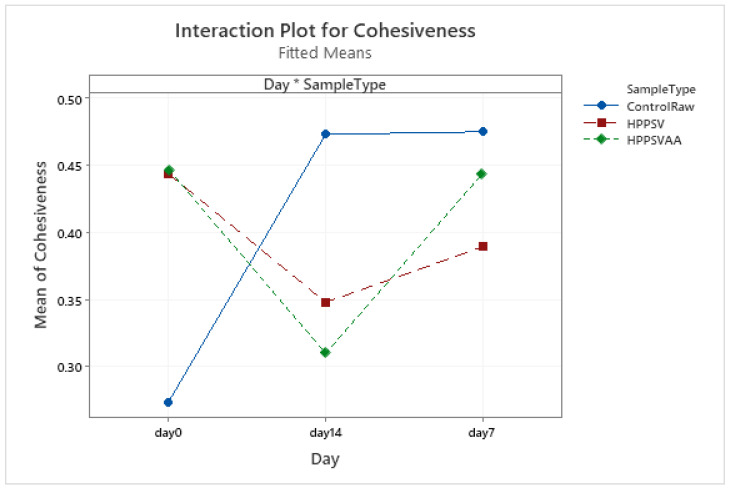
Effect of treatments and storage time on the cohesiveness of hybrid patty (HPPSV: HPP-SVCOOK).

**Figure A12:**
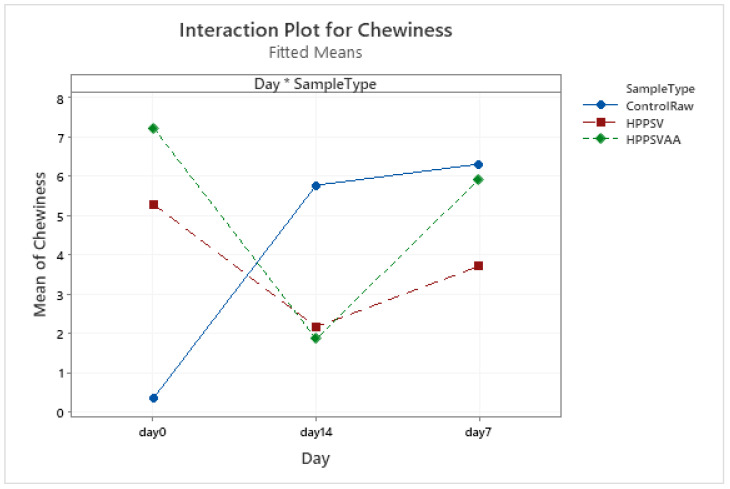
Effect of treatments and storage time on the chewiness of hybrid patty (HPPSV: HPP-SVCOOK).

In a previous study [20], it was noted that the changes in texture parameter of hybrid patties closely resembled the veal patties. Similarly, changes in the texture parameters of the hybrid product (Figure A9, Figure A10, Figure A11 and Figure A12) were similar to veal patties with the application of pressure and temperature, which might be attributed to the biochemical changes in meat during storage, pressure, and heat treatments [50,55,56], as explained in Section 3.3.1. All the measured texture parameters of the control followed a similar trend, with an increase over the first week of storage and no change over the rest of the storage days. Because no prominent effects of the treatments were observed in the plant-based patties, changes in the hybrid product conformed more to the veal product. The differences could be explained better by the changes observed in either plant-based or veal patties, as discussed earlier. 

### 3.4. Effect of Treatments and Storage Time on Color

#### 3.4.1. Veal Samples

**Figure A13:**
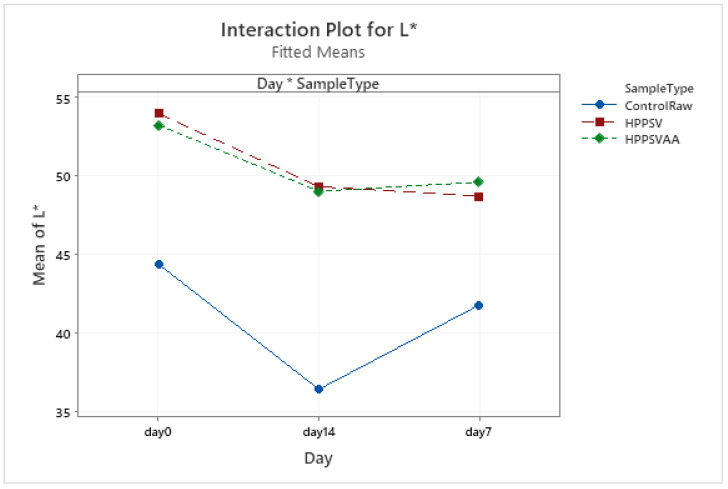
Effect of treatments and storage time on the L* coordinate of veal patty (HPPSV: HPP-SVCOOK).

Control and treated veal samples followed a similar trend in L* values where the maximum lightness was observed on the 0th day with a decline on the 7th day (Table 4, Figure A13). Compared to the HPP-SVCOOK treatment, the addition of carvacrol did not have a pronounced effect on the L* value. It could be inferred that the carvacrol dosage used in the current study was insufficient to change the L* value of patties.

**Figure A14:**
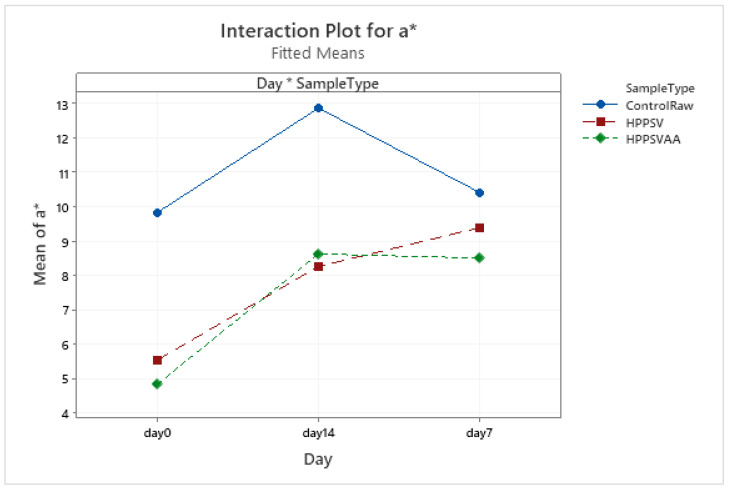
Effect of treatments and storage time on the a* coordinate of veal patty (HPPSV: HPP-SVCOOK).

The a* value of control samples increased with the increased number of storage days. The effect of HPP-SVCOOK led to a significant reduction (*p* < 0.05) in the a* value, while carvacrol had no observable effect on the redness value (Figure A14).

**Figure A15:**
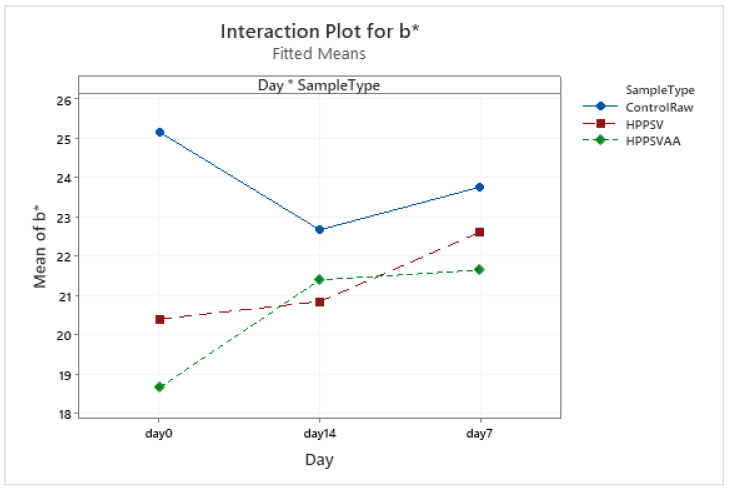
Effect of treatments and storage time on the b* coordinate of veal patty (HPPSV: HPP-SVCOOK).

The b* value of the control samples was the highest compared to the treated samples. On the 0th day, the effect of the HPPSV was prominent and significant (*p* < 0.05) in the b* values. In the case of samples treated with the antimicrobial agent, there was no significant change after the first week of storage (Table 4, Figure A15).

The effect of carvacrol on meat color has been inferred to be concentration-dependent. Lamb burgers formulated with carvacrol were found to have significant changes in color on 3 and 6 days of storage at a concentration of 1000 ppm of the antimicrobial agent with a limited effect at 300 ppm [30]; at the same time, in poultry patties and ground chicken enriched up to 300 ppm did not affect the color parameters [66,67]. Moreover, a higher L* and a lower a* value in HPP-treated meat and meat products have been previously noted. It might be because of the variation in myofibrillar packing and refraction of the sarcoplasm, which, in turn, leads to changes in the light scattering properties [48,68]. The changes in a* value can be because of the metmyoglobin formation [49,68]. Similar to our findings, a decrease in L* value of vacuum-packaged ground beef patties has been previously reported as a result of the deoxymyoglobin formation in the vacuum-packaged meat [54,69]. The deoxymyoglobin to metmyoglobin shift in stored meat might be a result of the pH changes and rise in the microbial load [54,70].

#### 3.4.2. Plant-Based Samples

On the 0th day, there was no significant difference between the treatments and control, whereas, on the 7th and 14th days, a significant difference (*p* < 0.05) was observed. 

**Figure A16:**
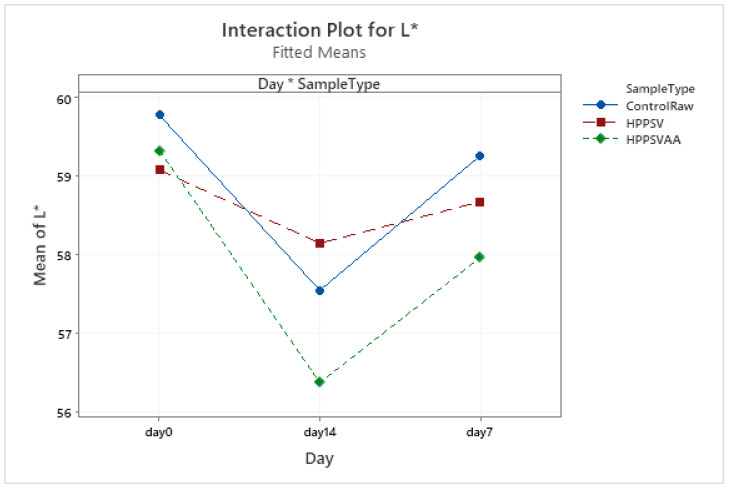
Effect of treatments and storage time on the L* coordinate of plant-based patty (HPPSV: HPP-SVCOOK).

It was seen that the L* values were reduced in the control and HPPSVAA samples with longer times of storage, whereas, in the HPP-SVCOOK samples, no significant reduction was noted. The lowest value for the L* value was observed in the samples with carvacrol (Figure A16). 

**Figure A17:**
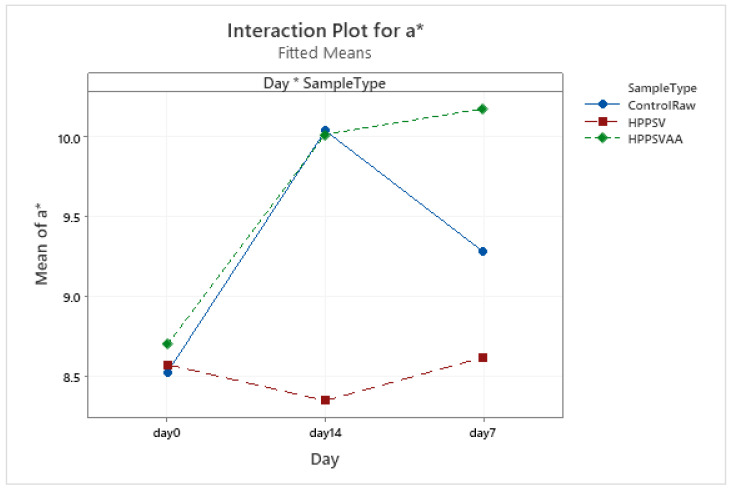
Effect of treatments and storage time on the a* coordinate of plant-based patty (HPPSV: HPP-SVCOOK).

**Figure A18:**
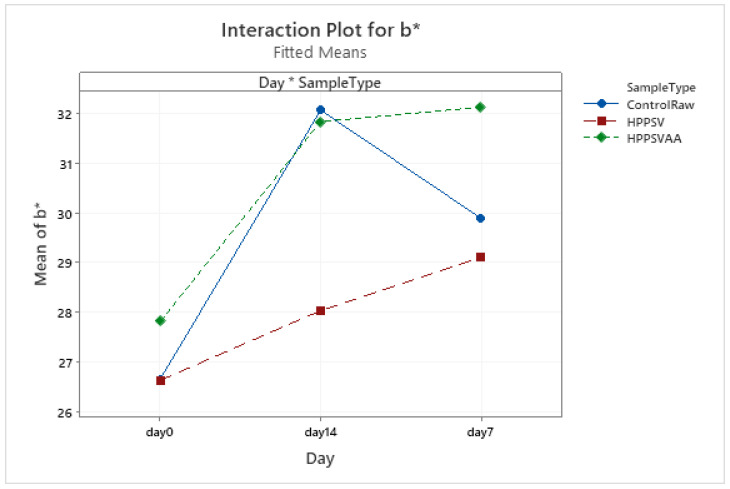
Effect of treatments and storage time on the b* coordinate of plant-based patty (HPPSV: HPP-SVCOOK).

In the case of a* values, a significant difference (*p* < 0.05) in the treatments was observed on the 7th and 14th days. The a* value of the HPP-SVCOOK samples remained consistent along the storage days (Figure A17). Similar trends were observed for the b* value too (Figure A18). A significant effect (*p* < 0.05) of the antimicrobial agent in the samples’ a* and b* values on the 7th and 14th day was observed compared to the pressurized sample without carvacrol (Table 4).

The characteristic color values can be inferred as a result of the original color of the plant-based raw material, and the product was observed to be relatively stable over the storage days, which coincides with other findings [20,71]. The color of plant-based products was not found to degrade rapidly, which might be attributed to the lack of oxidative deterioration of color as observed in meat products. The soy-based products naturally have a yellowish to beige color as opposed to the brown color of the cooked meat products. Heat-stable caramel colors, carotene, or heat-labile betanin, which are beetroot extracts that resemble raw and cooked meat, are added in commercial preparations. Moreover, the addition of colorants has not been a favorable solution to mimic the meat color in plant-based samples, due to the difference in the pH of the plant-based product and the optimal pH of the colorant [71]. The color changes in the control samples during storage can be caused by microbial contamination in the samples. 

#### 3.4.3. Hybrid Samples

**Figure A19:**
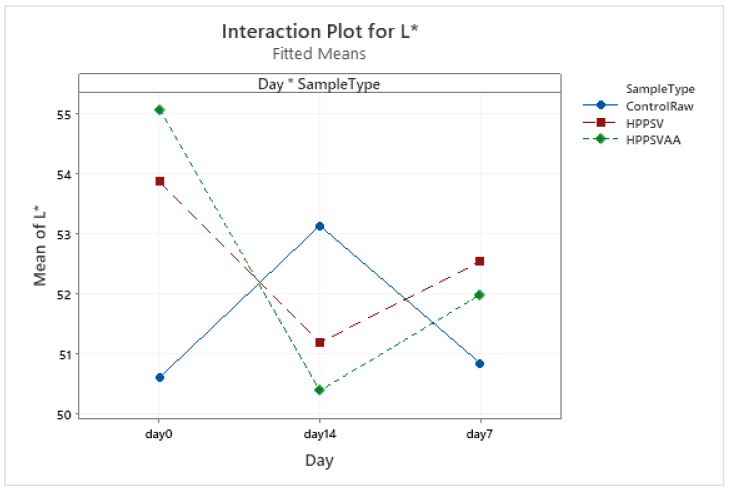
Effect of treatments and storage time on the L* coordinate of hybrid patty (HPPSV: HPP-SVCOOK).

**Figure A20:**
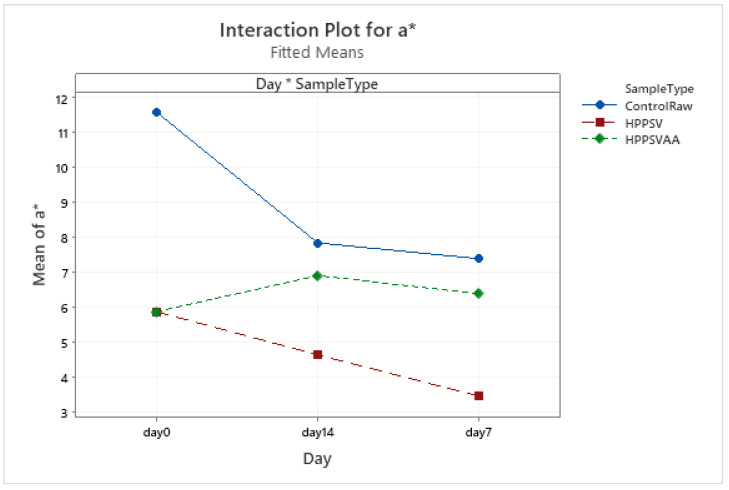
Effect of treatments and storage time on the a* coordinate of hybrid patty (HPPSV: HPP-SVCOOK).

It could be noted that there was no significant change in the L* value during the storage days nor due to the treatments (Figure A19). Interesting results were observed in the a* values. The HPP-SVCOOK and HPPSVAA samples had significantly lower (*p* < 0.05) a* values on the 0th day compared to the control. A significantly lower value (*p* < 0.05) was seen for the HPP-SVCOOK samples on the 7th day of storage (Figure A20). 

**Figure A21:**
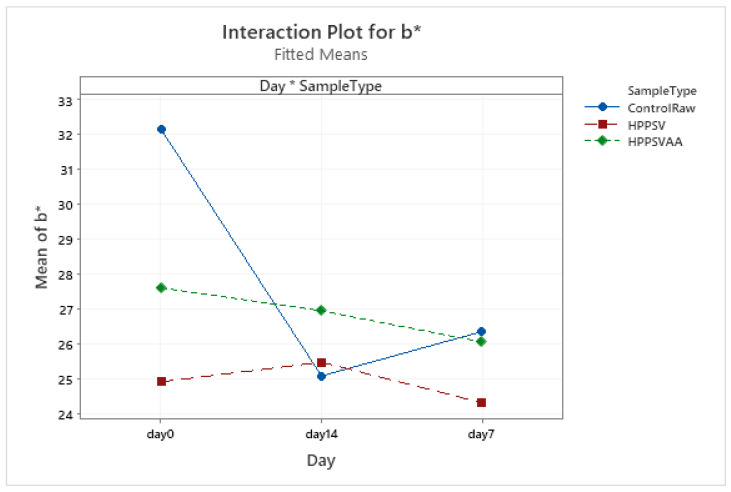
Effect of treatments and storage time on the b* coordinate of hybrid patty (HPPSV: HPP-SVCOOK).

In the case of control samples, a significant (*p* < 0.05) reduction in the b* value in the first week of storage could be seen. No significant change in the yellowness of the HPP-SVCOOK- and HPPSVAA-treated samples during the storage time was recorded (Table 4, Figure A21).

Buffalo meat sausage, an emulsion prepared with soy protein isolate, had marginal fluctuations in the color parameters with no definite trends during the 28-day storage period [65]. Changes in the color parameters can be attributed to the myofibrillar packing and metmyoglobin formation during the storage period in meat, which is detailed in Section 3.4.2 [48,68]. The effectiveness of preserving the a* value in the samples with carvacrol during the storage days might be a result of the antioxidant property of the antimicrobial compound [30]. The insignificant change in L* value was similar to the trend observed in the plant-based patty, which might be attributed to the mixed protein matrix in the hybrid patties. 

### 3.5. Microbial Counts

The microbial counts of samples at 0, 7, and 14 days of storage at 4 °C are shown in Table 5, Table 6 and Table 7. It should be noted that none of the batches tested and analyzed at all times during the study exhibited suspicious colonies of sulfite-reducing clostridia in TSN medium.

In regard to other microbial groups, the highest counts at day 0 were found in the control samples, with values around 5 log cfu/g for lactic acid bacteria and around 2 log cfu/g for molds and yeasts.

In the case of Enterobacteriaceae, plant-based and hybrid samples showed initial counts greater than 5 log cfu/g, 2 units of log more than the counts found in veal samples. These differences in Enterobacteriaceae counts could indicate a starting contamination of the plant material used for preparing the batches. For this reason, microbiological analysis of the plant-based raw material was carried out. This microbiological analysis showed the absence of microbial contamination (counts < 1 log cfu/g in all the culture media used). Therefore, accidental contamination during processing may explain the high Enterobacteriaceae counts found in the control batches. In any case, the HPP-SVCOOK application proved to be very effective from a microbiological point of view. The veal and hybrid samples subjected to HPP-SVCOOK presented a microbial load of 3 and 2 units of log cfu/g for lactic acid bacteria and molds and yeasts, respectively, significantly lower than their corresponding controls.

In the case of Enterobacteriaceae, HPP-SVCOOK was particularly effective because no Enterobacteriaceae (counts < 1 log cfu/g) were detected in any batch (including those with accidental Enterobacteriaceae contamination) after HPP-SVCOOK application. The fact that this treatment was more effective in eliminating Enterobacteriaceae than lactic acid bacteria could be attributed to the differences in the structure of their cell walls. Greater resistance to HPP treatments has been reported for Gram +ve bacteria (lactic acid bacteria) compared to Gram −ve (Enterobacteriaceae) [72,73]. A significant decrease in the a* value of the untreated hybrid samples was noted, which conforms toward a greenish shade, which might be caused due to the production of hydrogen sulfide and subsequent green sulfmyoglobin by the Enterobacteriaceae [74]. The significant increase in hardness observed in the control samples could be attributed to their higher microbial counts. Other workers have reported hardening in sausages due to microbial growth [65].

Incorporating carvacrol (as in HPPSVAA) does not seem to provide an additional microbial reduction to that of the HPP-SVCOOK treatment. The microbial counts in the HPPSVAA samples for all the microbial groups under study were very similar to those found in the batches with no antimicrobial agent.

Microbial counts increased in all samples during the 14 days of storage under refrigeration (Table 5, Table 6 and Table 7). This increase can be attributed to the development of the microorganisms that survived the HPP-SVCOOK and HPPSVAA treatments.

An increase in some batches of around 5 log units of the lactic bacteria indicated that it was best adapted to the storage conditions (vacuum packaging and refrigeration). Thus, the observed reduction in pH values during storage could be explained by the increased lactic acid bacteria. Other researchers have established a correlation between pH and growth of lactic acid bacteria [52]; current results coincide with their findings. In the case of veal samples where all the aerobic spoilage microflora growth was inhibited, the lactic acid bacteria became the major contributor to the microbial load, as seen in cured meats and vacuum-packaged beef. Some strains are known to cause souring, slime formation, and hydrogen sulfide production [75]. Initial lactic acid bacteria counts of less than 1 log cfu/g in vacuum-packaged and refrigerated cooked meat were found to reach 8 log cfu/g and cause spoilage during storage [76,77]. On the other hand, Enterobacteriaceae (whose initial counts had been very low in the batches subjected to HPP-SVCOOK or HPPSVAA treatment) and molds and yeasts (which do not seem to have adapted to the storage conditions) showed hardly any increase during the period studied.

## 4. Conclusions

The ready-to-eat veal, plant-based, and hybrid patties were found to be safe and shelf-stable throughout the studied storage period. This study noted no relevant effects of carvacrol on the microbiological and textural qualities of the veal, plant-based, or hybrid patties. The results establish that the use of combined emerging technologies could retain the quality of the meat and alternative protein products for the studied period of 14 days. Future studies on the fatty acid profile of the patties and antioxidant properties of carvacrol in HPP-SVCOOK products could be interesting. Further sensory analysis and market research need to be conducted on the HPP-SVCOOK-treated products to learn about the consumer acceptance as innovative, healthy, and sustainable meat and alternative protein products. These findings could help produce novel food products with longer safe storage periods, which can help bridge the food security and sustainability gaps. The agro-industries could meet the current market needs for alternative proteins without completely removing meat from the diet. It could help offer flexitarians and vegetarians more variety in their diet while being environmentally conscious. 

## Figures and Tables

**Table 1:** Proximate composition (mean value (standard deviation)) of the samples.

Sample	Moisture (%)	Protein (%)	Fat (%)	Ash (%)
V1	71.35 (0.12) ^a^	19.79 (0.11) ^ab^	6.13 (0.32) ^a^	2.48 (0.31) ^b^
V2	71.24 (0.02) ^a^	19.57 (0.01) ^ab^	7.20 (0.59) ^a^	2.45 (0.01) ^b^
PB1	57.41 (0.05) ^c^	19.80 (0.02) ^ab^	4.77 (0.24) ^a^	3.67 (0.00) ^a^
PB2	57.19 (0.01) ^c^	19.55 (0.29) ^ab^	7.88 (3.34) ^a^	3.62 (0.05) ^a^
H1	64.27 (0.54) ^b^	19.36 (0.13) ^b^	6.13 (0.15) ^a^	3.18 (0.00) ^a^
H2	64.38 (0.84) ^b^	20.06 (0.25) ^a^	5.65 (0.08) ^a^	3.19 (0.02) ^a^

V: veal patty, PB: plant-based patty, H: hybrid patty, 1: Control, 2: HPPSVAA. Mean values with different superscripts in the same column differ (*p* < 0.05).

**Table 2:** Effect of treatments and storage time on the pH (mean value (standard deviation)) of veal, plant-based, and hybrid patties.

Treatment	Storage Time (Days)	Veal Patty	Plant-Based Patty	Hybrid Patty
Control	0	5.86 (0.01) ^c^	6.94 (0.03) ^a^	6.39 (0.01) ^c^
	7	5.07 (0.01) ^f^	5.56 (0.02) ^c^	4.88 (0.02) ^f^
	14	5.04 (0.01) ^f^	6.02 (0.02) ^d^	4.98 (0.02) ^g^
HPP-SVCOOK	0	6.50 (0.01) ^b^	6.95 (0.02) ^a^	6.79 (0.05) ^a^
	7	5.75 (0.03) ^d^	6.05 (0.01) ^b^	5.90 (0.01) ^d^
	14	5.68 (0.01) ^e^	6.07 (0.02) ^b^	5.91 (0.01) ^d^
HPPSVAA	0	6.80 (0.03) ^a^	6.93 (0.03) ^a^	6.73 (0.01) ^b^
	7	5.72 (0.01) ^d^	6.07 (0.01) ^b^	5.88 (0.01) ^d^
	14	5.67 (0.01) ^e^	6.08 (0.02) ^b^	5.94 (0.01) ^e^

Mean values with different superscripts in the same column differ (*p* < 0.05).

**Table 3:** Effect of treatments and storage time on texture parameters (mean value (standard deviation)) of veal, plant-based, and hybrid patties.

Sample	Treatment	Storage Time (Days)	Hardness (N)	Springiness (N)	Cohesiveness	Chewiness
Veal	Control	0	2.92 (0.56) ^g^	0.42 (0.05) ^e^	0.36 (0.02) ^e^	0.44 (0.11) ^d^
		7	8.68 (2.23) ^fg^	0.61 (0.07) ^cd^	0.45 (0.09) ^bcd^	2.35 (0.70) ^cd^
		14	7.93 (1.39) ^efg^	0.61 (0.04) ^cd^	0.45 (0.04) ^bcd^	2.15 (0.35) ^cd^
	HPP-SVCOOK	0	32.32 (7.02) ^a^	0.77 (0.06) ^a^	0.49 (0.06) ^ab^	12.25 (3.09) ^a^
		7	12.02 (5.28) ^bc^	0.59 (0.06) ^abc^	0.38 (0.04) ^bc^	2.77 (1.66) ^b^
		14	21.36 (8.01) ^def^	0.69 (0.09) ^d^	0.46 (0.08) ^de^	7.11 (3.43) ^cd^
	HPPSVAA	0	28.00 (9.77) ^ab^	0.73 (0.14) ^ab^	0.56 (0.10) ^a^	11.69 (4.92) ^a^
		7	17.38 (8.97) ^cde^	0.66 (0.07) ^cd^	0.41 (0.08) ^bcd^	5.06 (3.37) ^bc^
		14	15.18 (7.17) ^cd^	0.62 (0.06) ^bcd^	0.45 (0.08) ^cde^	4.53 (2.78) ^bc^
Plant-based	Control	0	9.16 (2.06) ^c^	0.61 (0.09) ^a^	0.26 (0.03) ^bc^	1.52 (0.51) ^bc^
		7	9.37 (2.07) ^a^	0.49 (0.07) ^abc^	0.30 (0.03) ^ab^	1.39 (0.44) ^a^
		14	13.18 (1.74) ^c^	0.53 (0.08) ^bc^	0.28 (0.02) ^a^	1.97 (0.52) ^c^
	HPP-SVCOOK	0	11.62 (2.08) ^ab^	0.58 (0.08) ^ab^	0.28 (0.02) ^ab^	1.89 (0.51) ^ab^
		7	10.56 (1.42) ^bc^	0.51 (0.13) ^cd^	0.27 (0.03) ^bc^	1.43 (0.47) ^cd^
		14	10.60 (1.84) ^bc^	0.44 (0.06) ^bc^	0.27 (0.02) ^bc^	1.25 (0.30) ^c^
	HPPSVAA	0	9.11 (0.88) ^c^	0.35 (0.06) ^de^	0.25 (0.01) ^c^	0.79 (0.17) ^e^
		7	9.23 (1.74) ^bc^	0.29 (0.05) ^de^	0.24 (0.03) ^c^	0.67 (0.23) ^de^
Hybrid	Control	0	3.84 (0.88) ^e^	0.33 (0.07) ^d^	0.27 (0.06) ^e^	0.344 (0.11) ^d^
		7	18.16 (6.65) ^ab^	0.67 (0.06) ^ab^	0.47 (0.05) ^a^	5.77 (2.29) ^a^
		14	19.91 (4.17) ^ab^	0.67 (0.04) ^ab^	0.48 (0.04) ^a^	6.31 (1.31) ^ab^
	HPP-SVCOOK	0	17.01 (6.15) ^abc^	0.70 (0.05) ^a^	0.44 (0.07) ^ab^	5.29 (2.06) ^ab^
		7	12.17 (3.20) ^bcd^	0.50 (0.07) ^b^	0.35 (0.06) ^bc^	2.16 (0.90) ^bc^
		14	15.17 (3.52) ^cd^	0.62 (0.07) ^c^	0.39 (0.04) ^cd^	3.71 (1.16) ^cd^
	HPPSVAA	0	21.31 (8.40) ^a^	0.70 (0.11) ^a^	0.45 (0.07) ^ab^	7.23 (3.59) ^a^
		7	11.01 (3.30) ^ab^	0.52 (0.09) ^ab^	0.31 (0.03) ^ab^	1.86 (1.09) ^ab^
		14	20.30 (7.78) ^d^	0.64 (0.10) ^c^	0.44 (0.11) ^de^	5.92 (3.14) ^cd^

Values with different superscripts in the same sample column differ (*p* < 0.05).

**Table 4:** Effect of treatment and storage time on the color parameters of veal, plant-based, and hybrid patties.

Sample	Treatment	Storage Time (Days)	L*	a*	b*
Veal	Control	0	44.38 (2.86) ^c^	9.83 (1.04) ^bc^	25.15 (1.32) ^a^
		7	41.78 (2.75) ^c^	10.42 (1.47) ^bc^	23.75 (2.16) ^ab^
		14	36.42 (1.52) ^d^	12.88 (1.48) ^a^	22.66 (2.59) ^abc^
	HPP-SVCOOK	0	53.95 (1.85) ^a^	5.54 (0.58) ^e^	20.40 (0.10) ^cd^
		7	48.71 (1.66) ^b^	9.39 (1.13) ^bcd^	22.61 (1.61) ^bc^
		14	49.31 (1.65) ^b^	8.26 (0.91) ^d^	20.85 (0.99) ^cd^
	HPPSVAA	0	53.23 (2.18) ^a^	4.83 (0.79) ^e^	18.66 (2.68) ^d^
		7	49.60 (1.52) ^b^	8.51 (0.66) ^cd^	21.64 (1.66) ^bc^
		14	49.03 (1.05) ^b^	8.61 (0.75) ^cd^	21.39 (1.19) ^bc^
Plant-based	Control	0	59.77 (1.27) ^a^	8.52 (0.72) ^bc^	26.68 (1.04) ^e^
		7	57.54 (1.39) ^ab^	10.04 (0.64) ^ab^	32.08 (1.29) ^bc^
		14	59.25 (1.97) ^bc^	9.28 (0.93) ^a^	29.91 (1.36) ^a^
	HPP-SVCOOK	0	59.08 (0.95) ^ab^	8.57 (0.65) ^bc^	26.64 (1.39) ^e^
		7	58.15 (1.47) ^ab^	8.35 (0.60) ^bc^	28.04 (0.93) ^cd^
		14	58.68 (1.88) ^abc^	8.62 (0.75) ^c^	29.11 (1.49) ^cde^
	HPPSVAA	0	59.33 (1.33) ^ab^	8.70 (0.46) ^bc^	27.82 (1.96) ^de^
		7	56.38 (1.48) ^abc^	10.02 (0.62) ^a^	31.83 (1.22) ^a^
		14	57.97 (0.80) ^c^	10.18 (0.35) ^a^	32.13 (1.64) ^ab^
Hybrid	Control	0	50.60 (2.76) ^bc^	11.59 (1.66) ^a^	32.15 (1.76) ^a^
		7	53.13 (1.74) ^bc^	7.84 (0.51) ^bc^	25.09 (1.28) ^b^
		14	50.84 (3.74) ^abc^	7.39 (0.75) ^b^	26.36 (1.19) ^b^
	HPP-SVCOOK	0	53.87 (1.75) ^ab^	5.87 (0.98) ^de^	24.92 (3.55) ^b^
		7	51.18 (3.57) ^abc^	4.64 (0.96) ^f^	25.48 (3.18) ^b^
		14	52.54 (1.35) ^bc^	3.46 (1.28) ^ef^	24.32 (2.50) ^b^
	HPPSVAA	0	55.08 (1.78) ^a^	5.87 (0.89) ^de^	27.61 (2.01) ^b^
		7	50.38 (1.47) ^abc^	6.91 (0.84) ^cd^	26.95 (2.37) ^b^
		14	51.98 (1.54) ^c^	6.39 (0.90) ^bcd^	26.06 (2.67) ^b^

Values with different superscripts in the same sample column differ (*p* < 0.05).

**Table 5:** Lactic acid bacteria counts (mean of log cfu/g (standard deviation)) in the veal, plant-based, and hybrid patties during the 0, 7, and 14 days after processing.

		Time (Days)		
Sample	Treament	0	7	14
Veal	Control	5.29 (0.72) ^a^	6.07 (1.03) ^a^	6.43 (1.21) ^a^
	HPP-SVCOOK	2.06 (0.32) ^b^	1.92 (0.32) ^b^	4.55 (0.27) ^b^
	HPPSVAA	2.43 (0.28) ^b^	4.71 (1.19) ^a^	5.80 (0.36) ^ab^
Plant-based	Control	5.06 (0.47) ^a^	6.86 (0.98) ^a^	8.33 (0.94) ^a^
	HPP-SVCOOK	2.46 (0.14) ^b^	5.47 (0.96) ^a^	8.55 (1.03) ^a^
	HPPSVAA	2.53 (0.33) ^b^	4.93 (1.25) ^a^	7.96 (0.78) ^a^
Hybrid	Control	5.56 (0.65) ^a^	6.60 (0.85) ^a^	6.87 (1.01) ^a^
	HPP-SVCOOK	2.85 (0.21) ^b^	5.10 (1.02) ^a^	7.39 (0.67) ^a^
	HPPSVAA	2.46 (0.27) ^b^	5.07 (1.10) ^a^	7.63 (0.94) ^a^

Mean values with different superscripts in the same column differ (*p* < 0.05).

**Table 6:** Enterobacteriaceae counts (mean of log cfu/g (standard deviation)) in the veal, plant-based, and hybrid patty during the 0, 7, and 14 days after processing.

		Time (Days)		
Sample	Treatment	0	7	14
Veal	Control	3.08 (0.46) ^b^	3.62 (0.74) ^b^	4.53 (0.72) ^b^
	HPP-SVCOOK	<1 ^c^	1.21 (0.12) ^c^	<1 ^d^
	HPPSVAA	<1 ^c^	1.47 (0.21) ^c^	<1 ^d^
Plant-based	Control	5.69 (0.25) ^a^	6.23 (0.54) ^a^	7.01 (0.38) ^a^
	HPP-SVCOOK	<1 ^c^	1.47 (0.18) ^c^	1.57 (0.61) ^d^
	HPPSVAA	<1 ^c^	1.24 (0.17) ^c^	<1 ^d^
Hybrid	Control	5.53 (0.63) ^a^	5.80 (0.38) ^a^	6.10 (0.41) ^a^
	HPP-SVCOOK	<1 ^c^	<1 ^c^	3.51 (0.51) ^b^
	HPPSVAA	<1 ^c^	1.09 (0.08) ^c^	2.66 (0.65) ^c^

Mean values with different superscripts in the same column differ (*p* < 0.05).

**Table 7:** Fungi and yeast counts (mean of log cfu/g (standard deviation)) in the veal, plant-based, and hybrid patty during the 0, 7, and 14 days after processing.

		Time (Days)		
Sample	Treatment	0	7	14
Veal	Control	1.87 (0.45)	3.72 (0.32) ^b^	4.16 (0.59) ^c^
	HPP-SVCOOK	1.30 (0.21)	2.10 (0.27) ^c^	<1 ^e^
	HPPSVAA	1.30 (0.23)	1.90 (0.21) ^c^	<1 ^e^
Plant-based	Control	2.39 (0.88)	5.50 (0.71) ^a^	8.87 (0.85) ^a^
	HPP-SVCOOK	2.17 (0.62)	3.11 (0.31) ^b^	2.87 (0.43) ^d^
	HPPSVAA	2.17 (0.47)	3.00 (0.41) ^b^	<1 ^e^
Hybrid	Control	1.54 (0.14)	4.20 (0.61) ^b^	6.08 (0.71) ^b^
	HPP-SVCOOK	1.92 (0.37)	<1 ^d^	<1 ^e^
	HPPSVAA	1.69 (0.31)	2.22 (0.18) ^c^	<1 ^e^

Mean values with different superscripts in the same column differ (*p* < 0.05). Mean values in the same column bearing no superscripts do not differ (*p* > 0.05).

## Data Availability

Not applicable.
